# Tumor Genesis Syndrome Presenting as Severe Hypophosphatemia in a Patient With T-Cell Acute Lymphoblastic Leukemia

**DOI:** 10.7759/cureus.38815

**Published:** 2023-05-10

**Authors:** Osama N Dukmak, Mohammed Ayyad, Maram Albandak, Abdurrahman Hamadah, Kamel Gharaibeh

**Affiliations:** 1 Internal Medicine, Al-Quds University, Jerusalem, PSE; 2 Nephrology, St. Luke's Hospital, Duluth, USA; 3 Pulmonary and Critical Care Medicine, University of Maryland School of Medicine, Baltimore, USA

**Keywords:** tumor lysis syndrome, electrolyte disturbances, t-cell leukemia, hypophosphatemia, tumor genesis syndrome

## Abstract

Tumor lysis syndrome (TLS) is a medical emergency that can develop in leukemias and lymphomas as a first presentation or after the initiation of anti-neoplastic regimens. On the other hand, tumor genesis syndrome (TGS) is a rare condition associated with certain malignancies, especially those with a high neoplastic burden characterized by rapid proliferation, leading to avid uptake of phosphorus from the serum and culminating in hypophosphatemia. Interestingly, a combination of TLS and TGS can occur simultaneously in a subset of patients. This leads to the development of hypophosphatemia instead of the hyperphosphatemia commonly associated with TLS.

We herein present a case of severe asymptomatic hypophosphatemia in a patient with an incidental finding of T-cell acute lymphoblastic leukemia. The patient was initially diagnosed with TLS with hypophosphatemia, but further investigation revealed that the patient had isolated TGS.

## Introduction

Tumor lysis syndrome (TLS) is a tumor-related emergency that is commonly associated with the treatment of high-burden neoplasms such as acute leukemias and lymphomas [1]. The pathophysiology of this condition involves vigorous spontaneous and/or treatment-induced lysis of neoplastic cells. This leads to the release of intracellular content into the bloodstream, resulting in hyperuricemia, hyperphosphatemia, hyperkalemia, and hypocalcemia. These electrolyte abnormalities account for the major morbidity and mortality associated with this condition, leading to various, possibly fatal clinical manifestations, including acute kidney injury (AKI), seizures, arrhythmias, and multi-organ system failure [2].

On the other hand, tumor genesis syndrome (TGS) is another closely related condition encompassing a combination of electrolyte abnormalities, particularly hypophosphatemia, caused by neoplastic cytogenesis [3]. Similar to TLS, TGS most commonly occurs in neoplasms with high cellular burden and hematological malignancies such as acute leukemias and lymphomas. The most common electrolyte abnormality associated with TGS is hypophosphatemia; however, hypokalemia can also occur [4]. Of note, a subset of patients undergoing peripheral allogeneic stem cell transplant may exhibit a similarly marked uptake of phosphate during the engraftment phase [4].

We describe a case of acute T-cell lymphoblastic leukemia presenting with severe hypophosphatemia and hyperuricemia. The patient was initially diagnosed with TLS due to hyperuricemia. However, he did not fulfill the Cairo-Bishop criteria for laboratory TLS, and further investigations excluded pseudohypophosphatemia as well as gastrointestinal and renal losses. Ultimately, he was diagnosed with isolated TGS.

## Case presentation

A 41-year-old male patient presented to our hospital with a new onset headache, facial numbness, and fever. Past medical history was significant for long-standing hypertension, obstructive sleep apnea, and allergic rhinitis. Further history revealed a remote 10 pack-year smoking history but was otherwise normal. On physical examination, he was noted to have third cranial nerve palsy without evidence of lymphadenopathy or organomegaly. His vital signs were normal. Laboratory investigation revealed elevated serum creatinine (2.9 mg/dL), hypophosphatemia (0.6 mg/dL), hyperuricemia (13.2 mg/dL), elevated lactate dehydrogenase (LDH) (3880 IU/L), leukocytosis (137 × 10^9^/L), and thrombocytopenia (48 × 10^9^/L). Serum potassium level was normal (3.8 mEq/L), and ionized calcium level was on the upper limit of normal (10.8 mg/dL). Due to the patient’s blood findings, flow cytometry and bone marrow biopsy were done and confirmed the diagnosis of T-cell acute lymphoblastic leukemia (ALL).

Subsequently, he was treated with intravenous (IV) hydration, two sessions of leukapheresis, rasburicase, hydroxyurea, and allopurinol. Furthermore, the patient was started on a hyper-CVAD regimen (cyclophosphamide, vincristine sulfate, adriamycin, and dexamethasone) to treat his ALL. Following the cycles of the hyper-CVAD regimen, the patient underwent conditioning with cyclophosphamide and total-body irradiation followed by donor-matched allogeneic peripheral blood stem transplant.

One-month post-transplant, the patient developed skin and gastrointestinal manifestations consistent with graft versus host disease. Consequently, he was started on triamcinolone cream, prednisone taper therapy, and cyclosporine with significant improvement of his symptoms in the course of the ensuing few days to weeks.

On the 69th day post-transplant, the patient was hospitalized with relapsed T-cell ALL. His leukocyte count was elevated at 149.1 × 10^9^/L and consisted predominantly of blast cells (60%), which was then confirmed by flow cytometry. The patient was also found to have a repeatedly elevated serum creatinine level consistent with AKI, severe hypophosphatemia (<0.3 mg/dL), hyperuricemia (10 mg/dL), elevated LDH (1404 IU/L), and thrombocytopenia (82 × 10^9^/L). The laboratory findings are shown in Table 1. Urinalysis revealed a urine glucose level of 11 mmol/L (normal, 0-0.8 mmol/l), urine phosphorus less than 2 mmol/L (normal, 0.97-1.45 mmol/L), a phosphorus/creatinine ratio less than 0.06, indicating avid phosphorus reabsorption and excluding renal phosphate wasting as a cause, and a fractional excretion of sodium (FeNa) at 4.3% (normal, <1%), further suggestive of AKI. At that time, his vital signs and general physical examination were normal. The patient was empirically given rasburicase for his hyperuricemia. The nephrology team was then consulted and determined that he did not have TLS. Subsequently, aggressive IV fluid therapy and allopurinol were administered as prophylactic measures against TLS. Further management of AKI and severe hypophosphatemia were carried out under the supervision of the nephrology team.

**Table 1:** Summary of the patient’s laboratory following his second admission

Parameters	Patient’s findings	Normal value
Hemoglobin	10.3 g/dL	13.8-17.2 g/dL
White blood cell count	149.1 x 10^9^/L	4.5-11.0 x 10^9^/L
Platelet count	82 x 10^9^/L	150-500 x 10^9^/L
Creatinine	2.9 mg/dL	0.7-1.3 mg/dL
Potassium	4.6 mEq/L	3.5-5.0 mEq/L
Calcium	8.8 mg/dL	8.5-10.2 mg/dL
Phosphorus	0.2 mg/dL	2.8-4.5 mg/dL
Magnesium	1.7 mg/dL	1.7-2.2 mg/dL
Uric acid	10 mg/dL	3.5-7.2 mg/dL
Albumin	4.1 g/dL	3.4-5.4 g/dL
Lactate dehydrogenase	1404 IU/L	105-333 IU/L

**Figure 1:**
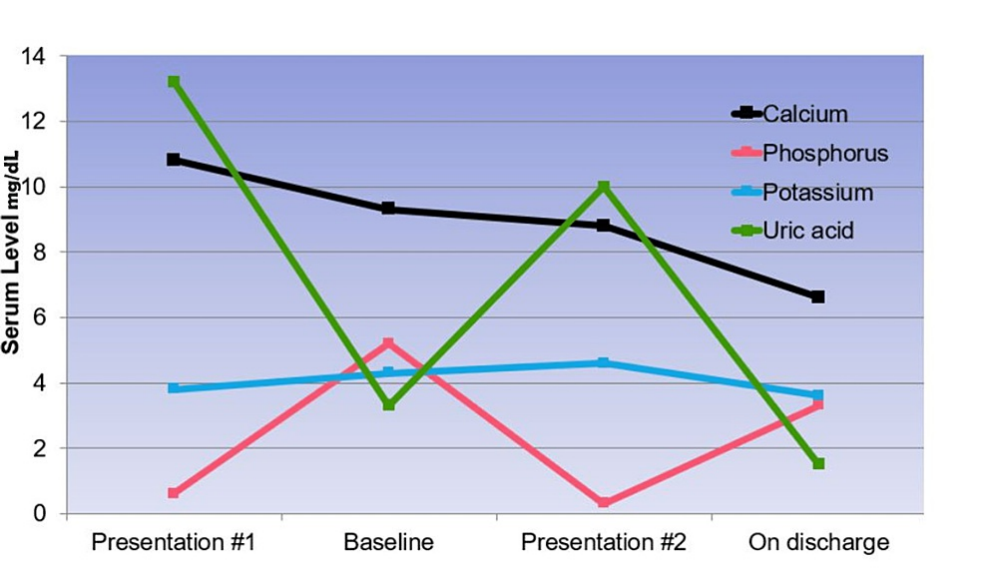
Serum electrolyte levels of the patient over the course of his illness

Based on our patient’s clinical and laboratory presentation, he was started on an aggressive oral and IV phosphorus replacement regimen. Given his high burden blast predominant leukocytosis, he underwent two sessions of leukapheresis followed by treatment with nelarabine and hydroxyurea. Following these interventions, the patient’s creatinine, uric acid, and leukocyte count improved, as shown in Figure 1. The patient continued to improve over the next few days with normalization of his kidney function and electrolyte levels. Ultimately, he was discharged with further follow-up at an outpatient clinic.

## Discussion

TLS is a medical emergency stemming from the release of intracellular components of tumor cells into the bloodstream due to the rapid turnover of tumor cells. It manifests with a constellation of metabolic derangements, including hyperkalemia, hyperuricemia, secondary hypocalcemia, and hyperphosphatemia, as the excretory capacity of the kidneys becomes overwhelmed. The combination of electrolyte derangements can potentially lead to cardiac rhythm disturbances and electrocardiogram (ECG) abnormalities, seizures, and neuromuscular weakness [5].

TLS has most commonly been reported following the induction of chemotherapeutic agents (58%), especially during the first week of chemotherapy, when high doses are utilized. It can also occur spontaneously prior to any treatment for tumor necrosis [6].

The typical phosphorus derangement found in TLS that is associated with hematological malignancies is hyperphosphatemia; however, severe hypophosphatemia has been described as well with a distinct etiology [7]. Interestingly, the term “tumor genesis syndrome” has been devised by Wollner et al. to describe the metabolic abnormalities that result from tumor growth [8]. Rapidly proliferating cancer cells increase their uptake of phosphorus in order to maximize their proliferative capacity. This provides the necessary phosphate for glycolytic intermediates in leukemic blast cells of myeloid origin, where oxygen fails to inhibit glycolysis. As a result, extracellular phosphate is depleted, leading to hypophosphatemia [9].

Severe hypophosphatemia is a life-threatening emergency that has also been described in patients with acute leukemia relapses, following allogeneic peripheral stem cell transplantation during hematopoietic reconstitution or following blastic transformation in chronic myeloid leukemia. However, increased phosphate consumption by rapidly proliferating tumor cells remains one of the most prevalent etiologies for hypophosphatemia in leukemic patients, which is considered an ominous sign that may portend cancer recurrence [9].

Hypophosphatemia from TGS must be differentiated from other etiologies of severe hypophosphatemia associated with neoplasms, namely tumor-induced osteomalacia. In this condition, increased production of fibroblast growth factor-23 leads to renal phosphate wasting, presenting similarly to hypophosphatemia associated with TGS [10].

Other causes of hypophosphatemia include low phosphate intake, urine and gastrointestinal losses, transcellular phosphate shifts from respiratory alkalosis, beta-adrenergic stimulation, refeeding syndrome, insulin infusion during the treatment of diabetic ketoacidosis, and hungry bone syndrome following parathyroidectomy [10]. Notably, cyclosporine (a calcineurin inhibitor) was found to cause phosphate (Pi) wasting by decreasing the gene expression of type II sodium/phosphate (Na/Pi) cotransporter in the proximal convoluted tubule, resulting in decreased phosphate reabsorption [11]. Extensive workup eliminated these etiologies as possible culprits in our patient. The workup included urine studies, which revealed avid renal reabsorption of phosphorus, excluding renal phosphate wasting secondary to cyclosporine therapy. Moreover, the patient’s dietary intake of phosphorus was normal, and intestinal losses from malabsorption, antacid, and diarrhea among other causes were excluded. Additional etiologies such as alkalemia, hyperglycemia, and hyperinsulinemia, which all cause intracellular phosphate shift, were excluded as well. Spurious hypophosphatemia was eliminated as a cause by serial phosphorus measurements using multiple platforms of Cobas and Vitros methods.

Eventually, the cause of hypophosphatemia in our patient was determined to be secondary to redistribution imbalance-induced hypophosphatemia. Elevated LDH and blast cells suggested that hypophosphatemia was a result of increased uptake of phosphorus by the rapidly proliferating neoplastic cells, consistent with TGS, which resulted from either rapid cellular uptake of phosphorus and/or rapid production of new cells that led to avid renal reabsorption of virtually all filtered phosphorus in urine, leading to hypophosphaturia. The patient was discharged home in a good status following the normalization of his laboratory abnormalities.

The Cairo-Bishop criteria are used for the classification of TLS into laboratory TLS (LTLS) and clinical TLS (CLTS). LTLS requires an abnormality in at least two or more of the variables presented in Table 2. Conversely, CTLS is confirmed by the presence of LTLS in addition to one of the following clinical manifestations: renal insufficiency, arrhythmia, seizures, or sudden death. In addition, other causes of AKI should be excluded [5]. In the presented case, in both admissions, our patient only exhibited elevated uric acid with normal calcium and potassium levels; thus, he did not satisfy the LTLS criteria. It is plausible that his elevated uric acid level was due to his underlying AKI given his elevated creatinine. As a result, he was promptly managed with the correction of the electrolyte derangements and aggressive IV hydration with notable improvement. In addition, allopurinol was given to prevent the development of TLS.

**Table 2:** Laboratory criteria for diagnosing tumor lysis syndrome

Variable	Change from baseline value	Value
Potassium	25% increase	≥6 mEq/L (or 6 mmol/L)
Phosphorus	25% increase	≥4.5 mg/dL (1.45 mmol/L)
Calcium	25% decrease	≤7 mg/dL (1.75 mmol/L)
Uric acid	25% increase	≥8 mg/dL (476 mmol/L)

Reviewing the literature, Chan et al. illustrated the uncommon presentation of severe hypophosphatemia, hypokalemia, acute renal failure, and acute respiratory failure in a 16-year-old patient with acute leukemia and marked leukocytosis [9]. On the other hand, Zakaria et al. described the case of a 14-year-old boy with acute T-cell lymphoblastic leukemia who had normal serum biochemistry except for marked hypophosphatemia and elevated LDH levels. Interestingly, the child had no symptoms related to low phosphate levels [12]. Moreover, Radi and Nessim described a case of severe hypophosphatemia in an 82-year-old patient with lymphoma. The authors determined the cause to be neoplastic intracellular uptake of phosphate [10]. Similarly, Aderka et al. presented a 49-year-old patient with acute myelogenous leukemia with hypokalemia, hypocalcemia, and severe hypophosphatemia (<1 mg/dL) manifesting as an extreme weakness [13]. The hypophosphatemia developed following the initiation of chemotherapy and lysis of blasts, mostly attributed to the excessive uptake of phosphate by the leukemic blasts [13]. Contrarily, our patient had normal potassium and calcium levels, and despite having a very low phosphate level, he did not exhibit signs of acute respiratory failure.

The previous cases highlight the variation in the clinical presentation and the electrolyte disturbances that manifest in TLS and TGS. We believe that TLS and TGS are parts of one spectrum, with hypophosphatemia being an ominous sign of aggressive tumor genesis and possible tumor recurrence, as evidenced in our case. Both conditions can occur independently or in combination with the same patient. The Cairo-Bishop criteria describe the typical electrolyte imbalances that occur in isolated TLS. However, if TLS occurs in combination with TGS, hypophosphatemia is a more likely presentation. Conversely, severe hypophosphatemia with evidence of hypokalemia may indicate early tumor recurrence in the setting of TGS [9]. Unfortunately, the Cairo-Bishop criteria are not comprehensive enough to encompass the diverse presentations and electrolyte imbalances that can arise from both of these conditions.

## Conclusions

TGS is a possibly life-threatening medical condition associated with rapidly proliferating neoplastic cells. The substantial neogenesis of tumor cells leads to vigorous intracellular uptake of phosphate, contributing to marked hypophosphatemia potentially leading to rapidly developing cardiorespiratory collapse. Interestingly, patients with high-burden malignancies, such as leukemias and lymphomas, can present with isolated TGS, isolated TLS, or combined TLS-TGS, denoting simultaneous rapid proliferation and destruction of neoplastic cells. This suggests that both conditions comprise parts of one spectrum, which can present within the context of a similar clinical presentation and a related pathophysiology. Due to the rapidly progressive and life-threatening nature of both conditions, physicians should maintain a high index of suspicion and pay meticulous attention to the ominous electrolyte perturbations. Particularly, the presence of hypophosphatemia in patients with hematological malignancies warrants further investigations to exclude renal, gastrointestinal, and intracellular shift as possible causes. Once these are excluded, TGS should be evaluated thoroughly and considered as the most likely culprit of hypophosphatemia.

## References

[1] Cairo MS, Bishop M (2004). Tumour lysis syndrome: new therapeutic strategies and classification. Br J Haematol.

[2] Howard SC, Jones DP, Pui CH (2011). The tumor lysis syndrome. N Engl J Med.

[3] Steiner M, Steiner B, Wilhelm S, Freund M, Schuff-Werner P (2000). Severe hypophosphatemia during hematopoietic reconstitution after allogeneic peripheral blood stem cell transplantation. Bone Marrow Transplant.

[4] Zamkoff KW, Kirshner JJ (1980). Marked hypophosphatemia associated with acute myelomonocytic leukemia. Indirect evidence of phosphorus uptake by leukemic cells. Arch Intern Med.

[5] Adeyinka A, Bashir K (2023). Tumor Lysis Syndrome. https://www.ncbi.nlm.nih.gov/books/NBK518985/#:~:text=Tumor%20lysis%20syndrome%20is%20most,syndrome%20are%20hepatoblastoma%20or%20neuroblastoma..

[6] Baudon C, Duhoux FP, Sinapi I, Canon JL (2016). Tumor lysis syndrome following trastuzumab and pertuzumab for metastatic breast cancer: a case report. J Med Case Rep.

[7] Imel EA, Econs MJ (2012). Approach to the hypophosphatemic patient. J Clin Endocrinol Metab.

[8] Wollner A, Shalit M, Brezis M (1986). Tumor genesis syndrome. Hypophosphatemia accompanying Burkitt's lymphoma cell leukemia. Miner Electrolyte Metab.

[9] Chan WK, Chang KO, Lau WH (2017). Tumour genesis syndrome: severe hypophosphatemia and hypokalemia may be ominous presenting findings in childhood acute myeloid leukaemia. Eur J Pediatr.

[10] Radi SA, Nessim SJ (2020). The case | severe hypophosphatemia in a patient with lymphoma. Kidney Int.

[11] Moz Y, Levi R, Lavi-Moshayoff V, Cox KB, Molkentin JD, Silver J, Naveh-Many T (2004). Calcineurin Aβ is central to the expression of the renal type II Na/Pi co-transporter gene and to the regulation of renal phosphate transport. J Am Soc Nephrol.

[12] Zakaria NH, Sthaneshwar P, Shanmugam H (2017). Severe asymptomatic hypophosphataemia in a child with T-acute lymphoblastic leukaemia. Malays J Pathol.

[13] Aderka D, Shoenfeld Y, Santo M, Berliner S, Shaklai M, Weinberger A, Pinkhas J (1980). Life-threatening hypophosphatemia in a patient with acute myelogenous leukemia. Acta Haematol.

